# Supplementary material for: Selective Inhibition of HDAC Class I Sensitizes Leukemia and Neuroblastoma Cells to Anticancer Drugs
Source: Biomedicines. 2021 Dec 6;9(12):1846. doi: 10.3390/biomedicines9121846 (PMC8698907; doi:10.3390/biomedicines9121846)
Supplement: Supplementary file 1 [file biomedicines-09-01846-s001.zip › Supplementary files/Supplementary.pdf]

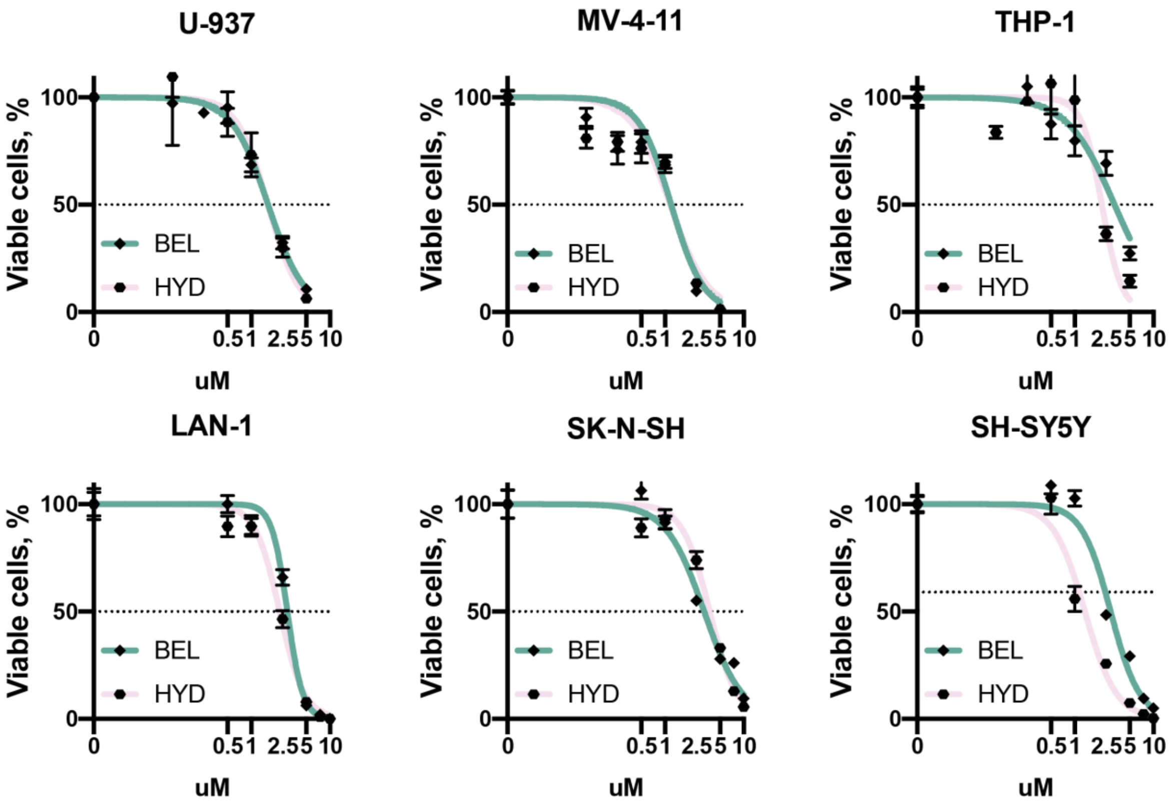

Figure S1. Viability of leukemia (U-937, MV-4-11, and THP-1) and NB (LAN-1, SK-N-SH, and SH-SY5Y) cells treated with belinostat (BEL, green) and hydrazostat (HYD, pink) relative

to DMSO-treated (control) cells. All experiments were performed in 3 replicates. Cells were counted by trypan blue exclusion on day 3 after the addition of inhibitors.

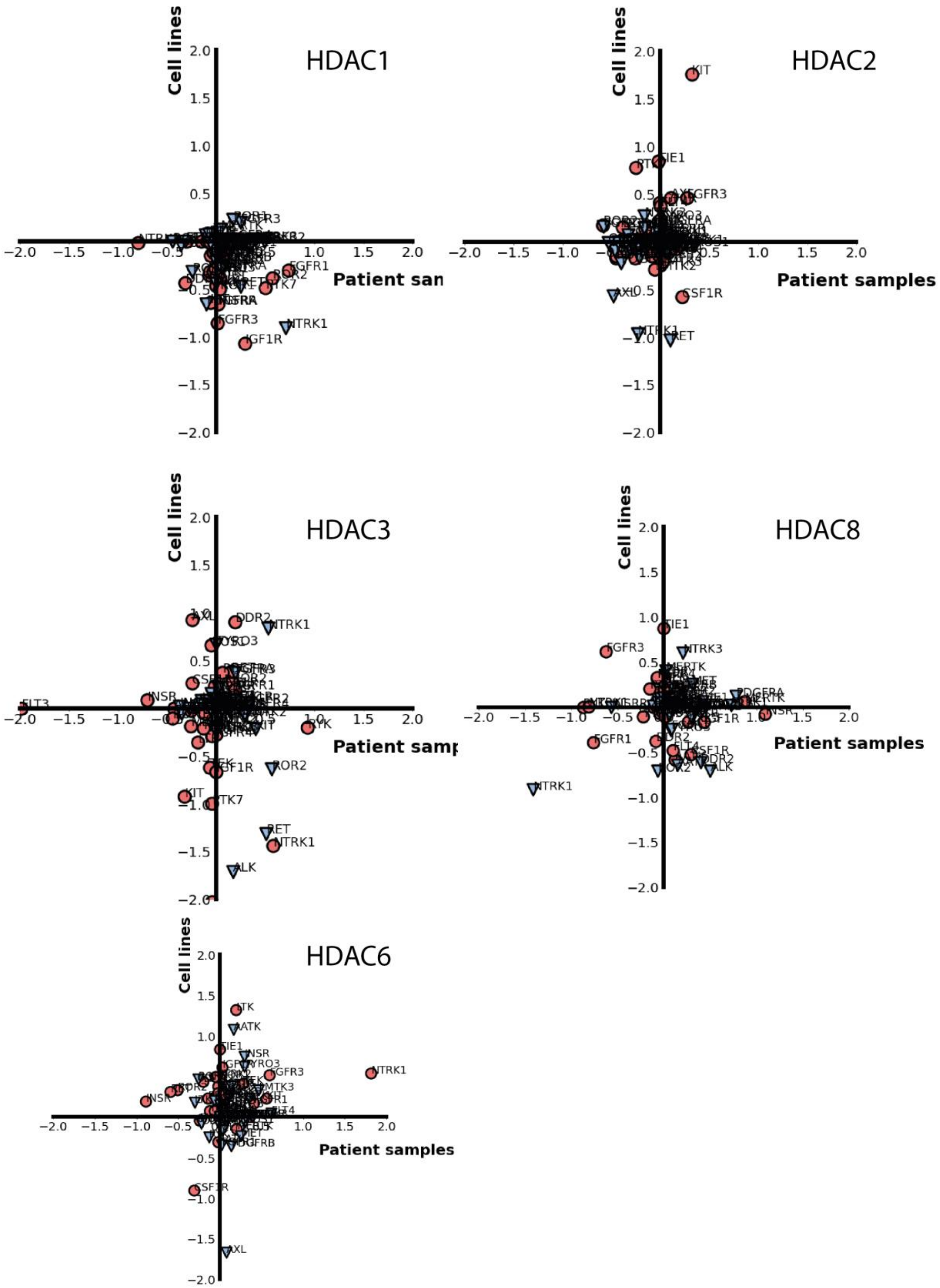

Figure S2. Elastic Net coefficients calculated for leukemia and NB samples (patients and cell lines), triangle – NB, round – leukemia.

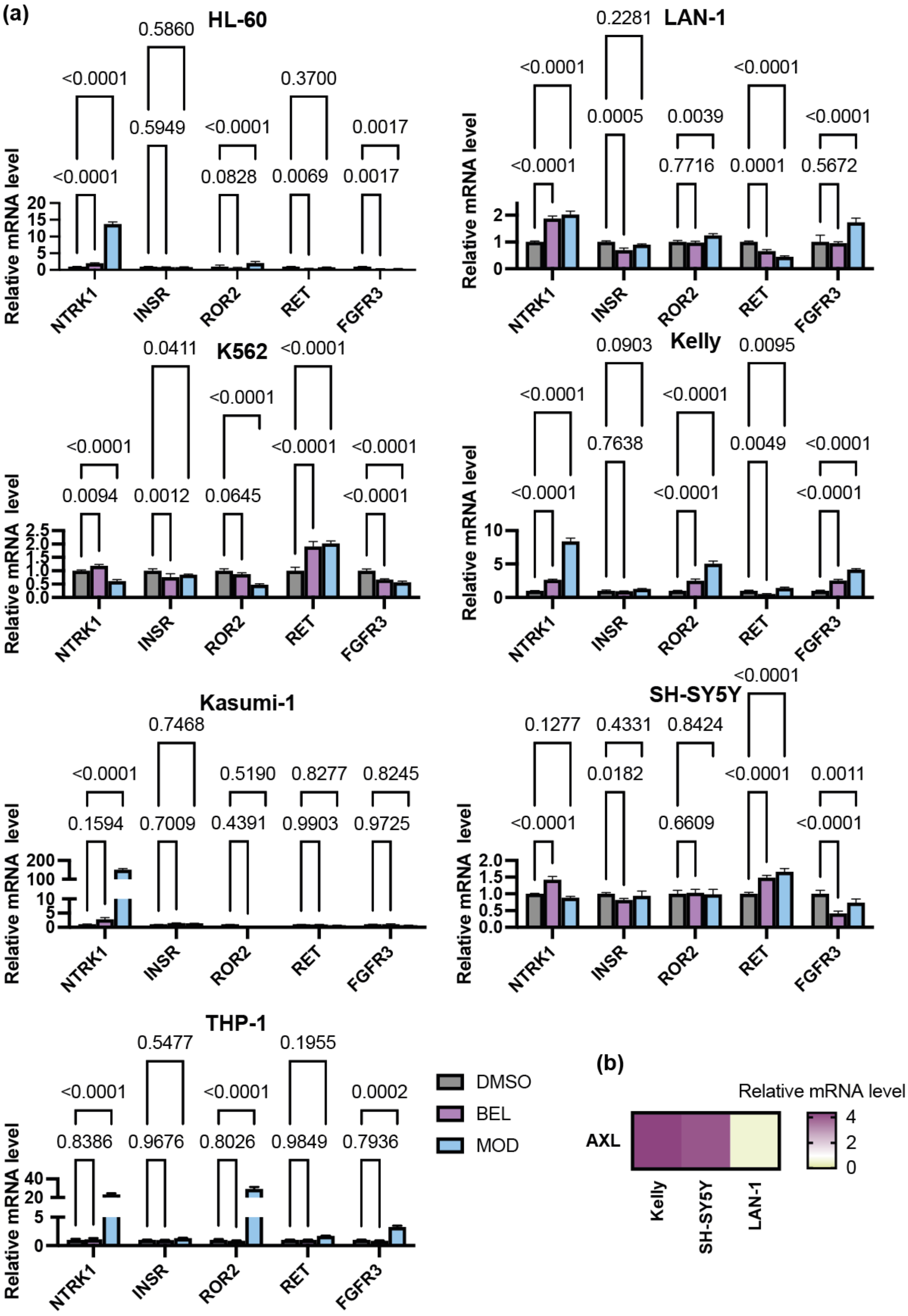

Figure S3. Expression of receptor tyrosine kinases coding genes at mRNA level in response to belinostat (BEL) and hydrazostat (HYD) measured by qRT-PCR.

(a) Fold change related to the expression in DMSO treated cells is shown.

(b) Relative mRNA level of *AXL* gene in NB cells. P-value was calculated by two-way ANOVA with no correction.

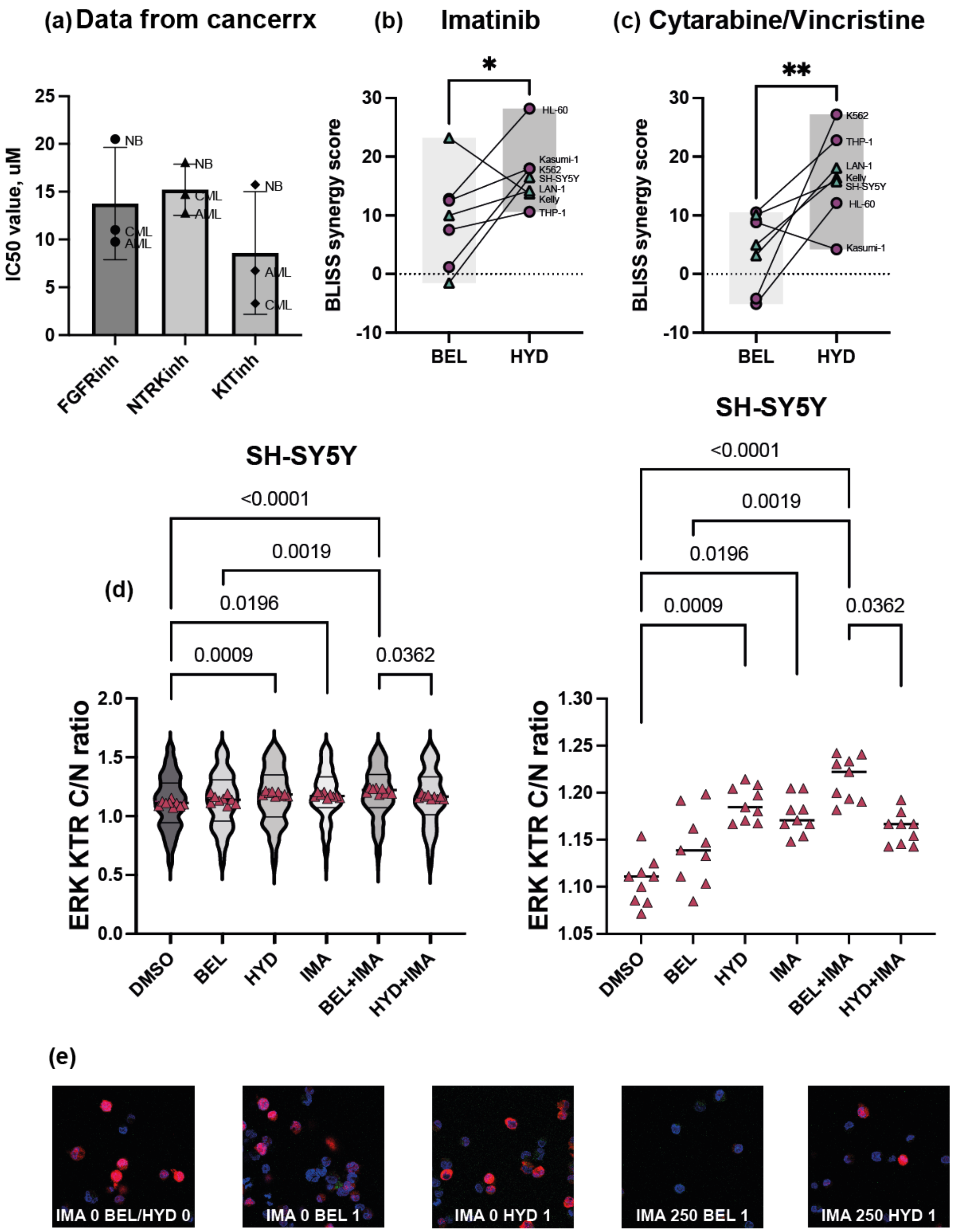

Figure S4. Sensitivity of leukemia and NB cells to RTK inhibitors and measurement of ERK activity.

(a) Geometric means of IC50 values (uM) of three FGFR-family (AZD4547, FGFR_3831, PD173074), two NTRK-family (AZD1332, GW441756) and three KIT, PDGFR, ABL1 inhibitors (imatinib, sunitinib, sorafenib) calculated for AML, CML and NB cells available at https://www.cancerrxgene.org.

(b) Comparison of BLISS synergy scores for imatinib with belinostat (BEL) or hydrazostat (HYD) – left panel; and for cytarabine with belinostat (BEL) or hydrazostat (HYD) – right panel. P-value was determined by Mann-Whitney t-test (* = p-value < 0.05, ** = p-value < 0.01).

(c) ERK activity in SH-SY5Y:ERK-KTR cells treated with BEL, HYD, IMA or their combinations determined as cytoplasm/nucleus fluorescence intensity of mClover protein. P-value was determined by Kruskal-Wallis test.

(d) Images of K562 cells obtained by confocal microscopy. Cells were incubated with anti-phospho-ERK antibodies (red), anti-KIT antibodies (green) and Hoechst (blue).

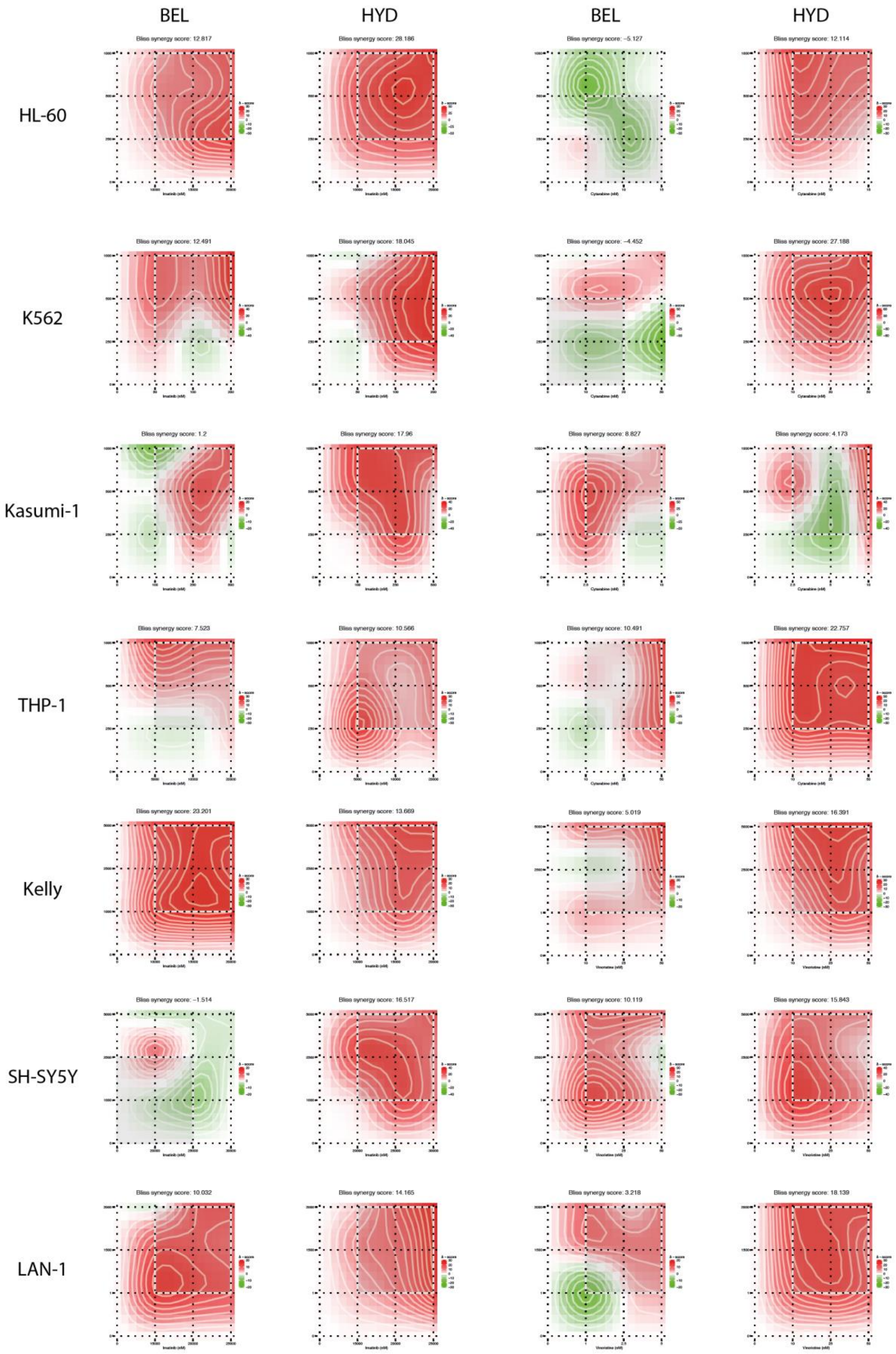

Figure S5. Viability of cells co-treated with imatinib and belinostat (BEL) or hydrazostat (HYD), cytarabine (leukemia), or vincristine (NB) with belinostat or hydrazostat. Bliss synergy

scores (SS) drug combinations are calculated with synergyfinder.fimm.fi and shown above each plot. The color corresponds to the synergy (red – area of synergy, green – area of antagonism).

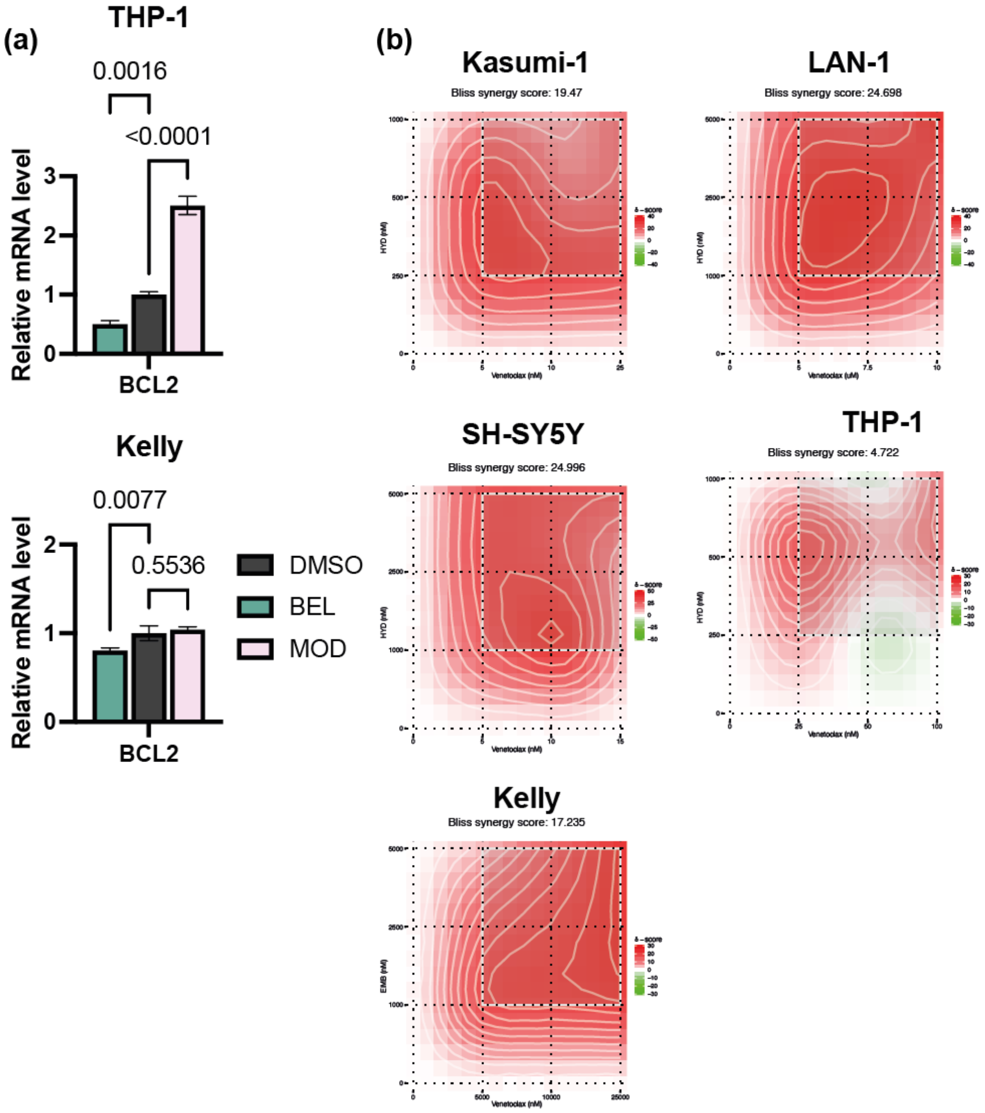

Figure S6. Hydrazostat enhances venetoclax toxicity.

(a) *BCL2* mRNA fold change in cells incubated with DMSO (grey), belinostat - BEL (green), or hydrazostat - HYD (pink) for 72h.

(b) Synergy plots obtained with synergyfinder.fimm.fi. The color corresponds to the synergy (red – area of synergy, green – area of antagonism). Bliss synergy scores (SS) drug combinations are calculated with synergyfinder.fimm.fi and shown above each plot. P-value was determined by one-way ANOVA with no correction.
